# Droplet etching of deep nanoholes for filling with self-aligned complex quantum structures

**DOI:** 10.1186/s11671-016-1495-5

**Published:** 2016-06-03

**Authors:** Achim Küster, Christian Heyn, Arne Ungeheuer, Gediminas Juska, Stefano Tommaso Moroni, Emanuele Pelucchi, Wolfgang Hansen

**Affiliations:** Institut für Nanostruktur- und Festkörperphysik, Center for Hybrid Nanostructures (CHYN), Universität Hamburg, Jungiusstraße 11, Hamburg, 20355 Germany; Tyndall National Institute, University College Cork, Lee Maltings Dyke Parade, Cork, T12R5CP Ireland

**Keywords:** Semiconductor, Nanostructuring, Self-assembly, Droplet etching, Quantum dot

## Abstract

Strain-free epitaxial quantum dots (QDs) are fabricated by a combination of Al local droplet etching (LDE) of nanoholes in AlGaAs surfaces and subsequent hole filling with GaAs. The whole process is performed in a conventional molecular beam epitaxy (MBE) chamber. Autocorrelation measurements establish single-photon emission from LDE QDs with a very small correlation function g ^(2)^(0)≃ 0.01 of the exciton emission. Here, we focus on the influence of the initial hole depth on the QD optical properties with the goal to create deep holes suited for filling with more complex nanostructures like quantum dot molecules (QDM). The depth of droplet etched nanoholes is controlled by the droplet material coverage and the process temperature, where a higher coverage or temperature yields deeper holes. The requirements of high quantum dot uniformity and narrow luminescence linewidth, which are often found in applications, set limits to the process temperature. At high temperatures, the hole depths become inhomogeneous and the linewidth rapidly increases beyond 640 °C. With the present process technique, we identify an upper limit of 40-nm hole depth if the linewidth has to remain below 100 *μ*eV. Furthermore, we study the exciton fine-structure splitting which is increased from 4.6 *μ*eV in 15-nm-deep to 7.9 *μ*eV in 35-nm-deep holes. As an example for the functionalization of deep nanoholes, self-aligned vertically stacked GaAs QD pairs are fabricated by filling of holes with 35 nm depth. Exciton peaks from stacked dots show linewidths below 100 *μ*eV which is close to that from single QDs.

## Background

**Fig. 1:**
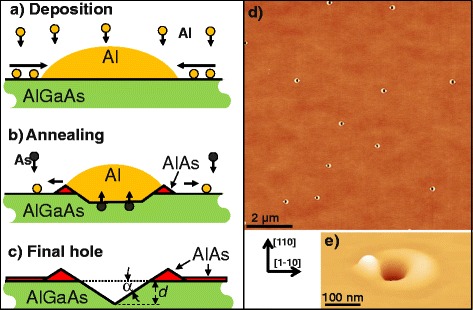
Self-assembly of nanoholes in AlGaAs surfaces by local droplet etching with Al. **a** Schematics of Al droplet formation during Al deposition. **b** Droplet etching by diffusion of As from the substrate into the droplet material during postgrowth annealing. **c** Final hole surrounded by an AlAs wall and planar AlAs layer caused by spreading of the initial droplet material. **d** AFM micrograph of an AlGaAs surface after Al droplet etching at temperature *T* = 630 °C, Al coverage *θ* = 1.8 ML, and As background pressure *F*_*As*_< 10 ^−7^ Torr. **e** Perspective and enlarged view of a nanohole from **d**

Local droplet etching (LDE) [1] has been established as a powerful extension of conventional molecular beam epitaxy (MBE) that allows the self-assembled fabrication of strain-free nanostructures. LDE was first demonstrated for drilling of nanoholes into GaAs or AlGaAs surfaces with Ga droplets as etchant [1–6]. Later, we have established also etching of AlGaAs and AlAs surfaces using LDE with Al droplets [7, 8] (Fig. 1). The usage of Al droplets is advantageous for quantum dot (QD) fabrication, since here, the walls around the nanohole openings (Fig. 1e) are composed of optically inactive AlAs [7].

We fill the nanoholes with GaAs to fabricate strain-free quantum dots (QDs) [7]. These QDs exhibit controlled and uniform size [7], clear excitonic features [8], and small excitonic fine-structure splitting [9]. So far, shallow nanoholes with depth of 10 to 15 nm are used as template for fabrication of QDs with narrow optical linewidth.

Recently, LDE of nanoholes with depth above 100 nm was demonstrated [10] by increasing the droplet material coverage *θ* and process temperature *T*. Such deep holes would allow the self-aligned fabrication of more complex coupled quantum structures stacked into a single hole. A prominent example are quantum dot molecules (QDM) where the nanoholes can be filled with two QDs separated by a tunnel barrier. QDMs are interesting, e.g., for studies of the tunnel coupling between quantum objects [11–15]. A further interesting example are hybrids composed of a single QD and a metallic nanostructure, which allow to study near-field radiative coupling between single-photon emitters and plasmonic modes [16, 17]. Here, nanoholes can be filled with a QD, a thin cap layer, and a metallic nanostructure.

As a precondition for the above functionalizations, the QD optical quality must be maintained also for filling of deep droplet-etched nanoholes. In a previous approach for deep nanohole creation, an optimized As background pressure of about 8 ×10^−7^ Torr, a droplet material coverage *θ* = 3.2 ML (monolayers), and a process temperature *T* = 620 °C were used. Nanoholes with 20–30 nm depth have been created [18] which is already sufficient for QDM fabrication. However, single QDs or stacked QD pairs filled into these nanoholes turned out to have very broad excitonic linewidth of several meV, i.e., more than one order of magnitude broader compared to QDs created in shallow holes [8]. Therefore, we study here different fabrication conditions with As pressure below 10^−7^ Torr and varied *θ* (1.0 Ml ≤*θ*≤ 2.4 Ml) as well as temperature *T* (600 °C ≤*T*≤ 690 °C). As a key result, we demonstrate the fabrication of single and vertically stacked QDs with linewidth below 100 *μ*eV using 35 nm deep LDE nanoholes as template.

## Methods

A schematic of a LDE process is sketched in Fig. 1. All samples are created on (001) GaAs wafers using solid source MBE. After growth of a 120 nm thick Al_*x*_Ga_1−*x*_As (*x* = 0.33) buffer layer, 1.0 ML up to 2.4 ML Al are deposited (Fig. 1a) at strongly reduced As pressure *F*_*As*_< 10 ^−7^ Torr and a process temperature varied from 600 up to 690 °C. The As pressure is at least hundred times smaller compared to typical MBE growth condition for planar GaAs or AlGaAs. The Al deposition results in the formation of low-density Al droplets (Fig. 1a) in Volmer-Weber growth mode [19]. During a subsequent annealing time *t* of 180 s, the initial droplets transform (Fig. 1b) into nanoholes surrounded by walls (Fig. 1c, e). Central processes for nanohole etching are the diffusion of As from the substrate into the liquid droplet material, a resulting liquefaction of the substrate at the interface to the droplet [20], and the spreading of the droplet material over the substrate surface [21]. An example of an AlGaAs surface with droplet etched nanoholes is shown in Fig. 1d, e. The structural features of the nanoholes are characterised using atomic force microscopy (AFM) in tapping mode.

The basic optical characterization of single QDs is done with microphotoluminescence spectroscopy (PL) at liquid Helium temperature. Individual QDs are selected using a focused helium-neon laser (*λ* = 632.8 nm) or a green diode laser (*λ* = 532 nm) for excitation. A combination of grating spectrometer and liquid nitrogen cooled CCD is used to disperse and detect the luminescence signal. The resolution limit of this setup is about 40 *μ*eV. The measurements of the exciton fine-structure splitting (FSS) were taken using a combined measurement and fitting procedure [22, 23]. A linear polarizer was placed at the entrance of the spectrometer, and linear polarization components were analyzed by rotating emitted light with a half waveplate installed before the polarizer. A set of measured exciton spectra were fitted by the Lorentzian function and the peak centers plotted as a function of a half-waveplate angles. The amplitude of the obtained sinusoid corresponds to the value of FSS.

Autocorrelation curve was measured using a standard Hanbury Brown and Twiss setup: exciton transition was filtered by the monochromator, split by a non-polarizing 50:50 beamsplitter and sent to two avalance photo diodes (APD) coupled to multimode fibers. One APD was used to start the photon counting module, the other, delayed, to stop: an obtained histogram of the measured time intervals is directly proportional to the second-order correlation

## Results and discussion

### Nanohole uniformity

**Fig. 2:**
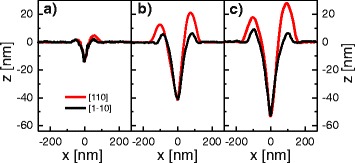
Depth profiles along [110] and [1$\bar 1$0] direction of Al LDE nanoholes etched into AlGaAs at: *θ* = 1.0 ML, *F*_*As*_< 10 ^−7^ Torr, and varied **a** *T* = 600 °C, **b** *T* = 630 °C, **c** *T* = 660 °C

Process parameters influencing the hole depth are the As background pressure [24], the surface coverage *θ* with deposited Al droplet material [10], and the process temperature *T* during LDE [10]. At minimized As flux, the hole depth *d* can be estimated from a simple scaling law [10]:

1 $$ d = c_{h} \theta^{2/3} \exp [- E_{h} /(k_{B} T)]  $$

with constants *c*_*h*_ = 1.5 ×10^11^ nm ML ^−2/3^ and *E*_*h*_ = 1.73 eV. For illustration, Fig. 2a–c shows typical profiles of nanoholes etched at *T* = 600 °C, *T* = 630 °C, and *T* = 660 °C, respectively. It is clearly discernible that the hole depth is increasing with *T*.

**Fig. 3:**
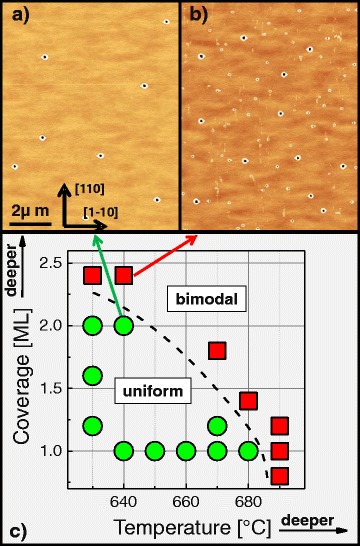
**a** AFM image of an AlGaAs surface with uniform nanoholes after LDE at *T* = 640 °C, *θ* = 2.0 ML, and *F*_*As*_< 10 ^−7^ Torr. **b** AFM image of an AlGaAs surface with bimodal nanoholes after LDE at *T* = 640 °C, *θ* = 2.4 ML, and *F*_*As*_< 10 ^−7^ Torr. **c** Phase diagram of uniform or bimodal hole generation dependent on process temperature and Al coverage. *Green circles*indicate uniform and red squares bimodal holes. As a guide to the eye, we drew a *dashed line* between the *red squares* and *green circles*

Figure 3a shows an AlGaAs surface with nanoholes etched at *T* = 640 °C and *θ* = 2.0 ML. The nanoholes have a uniform size distribution with hole depth varying by ±10 *%*. In contrast, a sample with increased *θ* = 2.4 ML shows a non-uniform bimodal hole-size distribution (Fig. 3b). The transition from uniform to non-uniform holes with increasing droplet material coverage was already reported previously [10] and attributed to a change of the surface reconstruction. Here, we observe in addition a uniform to non-uniform transition for an increase of the process temperature. Figure 3c summarizes the results and indicates a borderline between uniform and bimodal hole formation related to an increase of both, the temperature as well as the Al coverage. Obviously, the fabrication of uniform QDs requires the process temperature to be below a coverage dependent maximum value.

### Quantum dots optical properties

For quantum dot fabrication, the LDE nanoholes are partially filled by deposition of a GaAs amount corresponding to a 0.5 nm thin GaAs layer on a plane (001) AlGaAs surface. For the present QDs, we determine heights of 5–7 nm by assuming that the part of the GaAs flux impinging on the area of the nanohole opening migrates downwards and fills up the hole starting from its bottom [7]. In detail, the thickness of the GaAs filling layer and the hole opening diameter define the size of the GaAs QDs. The such determined height agrees with AFM measured profiles, where a large number of unfilled nanoholes is compared to holes after filling with QDs. Finally, the QDs are capped with 80 nm AlGaAs.

**Fig. 4:**
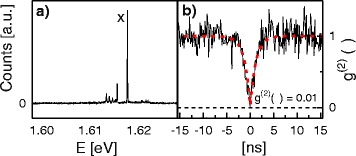
**a** PL spectrum of a QD used for the autocorrelation measurement. **b** Autocorrelation measurement (*line*) of the exciton peak from the *dot* in **a** together with a fit (*red dashed line*). The g ^(2)^(0) value is 0.01

So far, we have used 10–15 nm deep holes for the fabrication of GaAs QDs with narrow optical linewidth [8]. Figure 4a shows a PL spectrum with narrow exciton (*X*) peak from a single QD. The QD was filled into a 15-nm-deep hole which is fabricated using LDE at *T* = 600 °C and *θ* = 1.0 ML. Autocorrelation measurements of this QD are performed in order to proof single-photon emission. The exciton-peak correlation function g ^(2)^(*τ*) is plotted in Fig. 4b as function of *τ*. The data are fitted using 1−*α* exp(−*τ*/*t*_*d*_), with decay time *t*_*d*_ = 1.18 ns and *α* = 0.99 being fit parameters. The very low g ^(2)^(0) = 0.01 clearly indicates single-photon emission and non-classical photon antibunching [25].

**Fig. 5:**
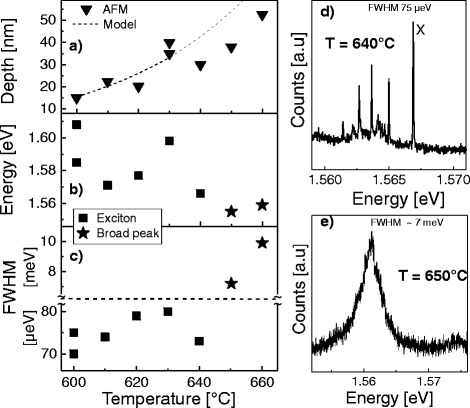
Influence of the temperature during LDE for *θ* = 1 ML and *F*_*As*_< 10 ^−7^ Torr on **a** the average depth of the initial holes. The model results are calculated using Eq. 1, **b** the average energy of the QD ground-state emission, and **c** the average full width at half maximum (FWHM) of the exciton peak or the broad peak for *T*≥ 640 °C. Note the change of the scale indicated by the *dashed line*. **d** Typical single-dot PL spectrum with exciton (*X*) peak and higher excitonic complexes of a sample etched at *T* = 640 °C. The exciton peak has a FWHM of 75 *μ*eV. **e** Typical single-dot PL spectrum with broad peak of a sample etched at *T* = 650 °C. The broad peak has a FWHM of about 7 meV

In order to study QD formation also in deeper holes, we have fabricated a QD sample series with varied temperature during the LDE process from *T* = 600 up to 660 °C. The Al coverage *θ* = 1.0 ML guarantees a uniform size distribution. Figure 5a shows the hole depth increasing from 15 up to 53 nm with increasing *T* in quantitative agreement with the model predictions of Eq. 1. The average QD ground-state emission shows a slight red-shift with increasing *T* (Fig. 5a). This red-shift might be related to the larger hole opening at higher *T* and the, thus, larger amount of filling material forming the dot.

As the most important finding here, Fig. 5c demonstrates that the requirement of small luminescence linewidth sets a further upper limit to the process temperature. For temperatures up to 640 °C, the single-dot PL spectra show a clear exciton (*X*) peak together with higher excitonic complexes at lower emission energy. An example is plotted in Fig. 5d. The linewidth of the exciton peaks is 70–80 *μ*eV in the temperature range of *T* = 600–640 °C. An only slight increase of the process temperature to 650 °C yields QDs with substantially different optical properties. Now, the single-dot PL spectra show a very broad peak with linewidth of several meV. A typical spectrum with such broad PL peak is shown in Fig. 5e. We assume that the exciton and biexciton lines are strongly broadened and merge to one broad peak. From this broad peak, we cannot determine the linewidth of the individual excitonic lines. The linewidth is rather determined for the overall peak, marked as stars in Fig. 5c. We note that this abrupt PL peak broadening is in contrast to the continuous behavior of the hole depth and ground-state energy (Fig. 5a, b). Also, the shape of the nanoholes as recorded before GaAs deposition shows no significant changes between *T* = 630–660 °C (Fig. 2b, c). So far, the reason for the PL peak broadening is not clear. We speculate that the abrupt peak broadening might be caused by a material decomposition at high process temperatures.

In summary, the above results suggest that LDE temperatures above 640 °C should be avoided for sharp PL peaks. This corresponds to a maximum hole depth of about 40 nm (Fig. 5a).

**Fig. 6:**
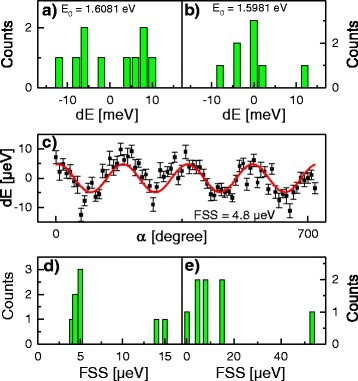
Distribution of exciton peak energies *E*_0_+*d* *E* for a number of QDs **a** of type I (fabricated by filling of about 15 nm deep holes) and **b** of type II (fabricated by filling of about 35 nm deep holes). **c** Example of a polarization angle *α*-dependent measurement (*symbols*) of the energetic position dE+1616342.2 *μ*eV of the QD exciton emission. A sinus-type fit (*line*) agrees with a neutral exciton fine-structure splitting (FSS) of 4.8 *μ*eV. **d** Neutral exciton fine-structure splitting (FSS) for a number of type I QDs and **e** for type II QDs

In Fig. 6a, b, the distribution of the exciton energy of QDs formed in about 15 nm deep holes (type I, *T* = 600 °C, *θ* = 1.0 ML) and about 35 nm deep holes (type II, *T* = 630 °C, *θ* = 1.0 ML) is compared. Both samples have a similar behavior with an energy spread of about ±10 meV.

Figure 6c shows an example of a polarization angle-dependent measurement of the QD exciton emission energy. From a sinus-type fit, a neutral exciton FSS of 4.8 *μ*eV is determined. In Fig. 6d, e, the FSS from several type I and type II QDs are compared. For type I QDs, the data show a few QDs with FSS larger than 10 *μ*eV and a bunching of FSS values around a mean value of 4.6 *μ*eV. For type II dots, the FSS values are more widely spread with one QD having a large FSS of 53 *μ*eV and a bunching around a mean FSS value of 7.9 *μ*eV. The increasing FSS for the type II dots might be caused by a stronger asymmetry [26] of the deeper holes. These data suggests the present QDs for the field of quantum cryptography, where a small FSS is a key prerequisite for the generation of entangled photons [27, 28].

### Vertically stacked quantum dots

**Fig. 7:**
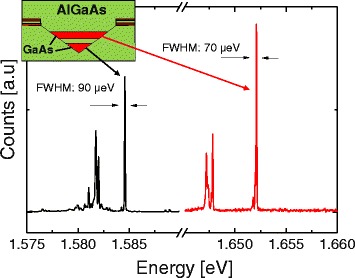
Low-temperature PL spectra from two stacked QDs forming a QD pair at very low excitation power of 0.1 W/cm^2^ (*first dot*) and 0.2 W/cm^2^ (*second dot*). The *black line* represents the emission from the first and the *red line* from the second QD. The nanohole template was fabricated at *T* = 630 °C, *θ* = 1.0 ML, and *F*_*As*_< 10 ^−7^ Torr. The *inset* shows a schematics of a self-aligned QDM

As a demonstration for the improved possibilities of a deep hole template, we use the above type II process conditions to fabricate vertically stacked quantum dot pairs. In detail, we fill 35 nm deep nanoholes with a GaAs QD, an AlGaAs or AlAs tunnel barrier, a second GaAs QD, and finally, an AlGaAs cap layer. A schematics of such a self-aligned QDM is shown as inset in Fig. 7. The deposited tunnel barrier layer thickness is nominally 3 nm.

A comparison of AlGaAs and AlAs tunnel barriers yields much smaller linewidth for samples with an AlAs barrier. QD pairs with AlGaAs tunnel barrier have typically a linewidth of several 100 *μ*eV, whereas those with an AlAs barrier have linewidth below 100 *μ*eV. As an example, a PL spectrum of a QD pair with AlAs barrier is plotted in Fig. 7. We can clearly separate emission peaks from the first (bottom) and the second (top) dots. The first QD has a ground-state emission at 1.585 eV which agrees with the emission energy of a single type II QD. The difference between p- and s-shell emission is 11.7 meV, which is much smaller than the energy separation of 68 meV between both QDs. Our identification is supported by the systematic behavior of the emission energies in samples prepared with different filling pulse numbers. If the filling of the first dot is kept constant and the filling of the second dot is increasing the high-energy luminescence lines shift to lower energy. The second dot emits at significantly higher energy 1.652 eV which is caused by the smaller extent in growth direction and the, thus, higher quantization energy. The energy difference between both dots can be adjusted by the respective nanohole filling levels. For both QDs, the exciton peaks have the highest energy, while multiexcitonic peaks are red-shifted. As most important point here, the FWHM of the excition lines are 90 and 70 *μ*eV for the first and second dots, respectively. This indicates that QD pairs fabricated by filling of LDE nanoholes show no additional peak broadening in comparison to single QDs. Furthermore, the linewidths are sufficiently small for investigations of molecule-resonant states in such quantum dot molecules.

Additional experiments, not shown here, use a gate voltage for tuning the energy levels of a QD pair into resonance. The data exhibit anticrossings, which is a clear proof for tunnel coupling and the presence of molecule-resonant states.

## Conclusions

With our results, we expand the possibilities of the local droplet etching method. So far, nanoholes with depth of 10–15 nm have been used as template for fabrication of QDs with narrow PL linewidth. This work demonstrates the generation of uniform QDs with narrow exciton peak linewidth below 100 *μ*eV also in up to 40 nm depth holes. The deeper holes allows filling with more complex heterostructures like coupled quantum systems. As a first application of the deep droplet-etched nanoholes, self-aligned vertically stacked quantum dot pairs are successfully fabricated by hole filling. The quantum dot pairs establish exciton peaks with small linewidths of 70–90 *μ*eV which is close to that from separated QDs. The narrow linewidths allow studies of molecule resonances in these systems. As an important advantage of the present method, the size of the individual QDs as well as the tunnel barrier thickness can be adjusted individually by the respective filling layer.
